# Beneficial Effects of *Manilkara zapota*-Derived Bioactive Compounds in the Epigenetic Program of Neurodevelopment

**DOI:** 10.3390/nu16142225

**Published:** 2024-07-11

**Authors:** Cristina Russo, Maria Stella Valle, Floriana D’Angeli, Sofia Surdo, Salvatore Giunta, Antonio Carlo Barbera, Lucia Malaguarnera

**Affiliations:** 1 Section of Pathology, Department of Biomedical and Biotechnological Sciences, School of Medicine, University of Catania, 95123 Catania, Italy; cristina.russo@unict.it (C.R.); lucmal@unict.it (L.M.); 2 Section of Physiology, Department of Biomedical and Biotechnological Sciences, University of Catania, 95123 Catania, Italy; 3 Department of Human Sciences and Quality of Life Promotion, San Raffaele Roma Open University, 00166 Rome, Italy; floriana.dangeli@uniroma5.it; 4 Italian Center for the Study of Osteopathy (CSDOI), 95124 Catania, Italy; s.surdo98@yahoo.com; 5 Section of Anatomy, Department of Biomedical and Biotechnological Sciences, University of Catania, 95123 Catania, Italy; sgiunta@unict.it; 6 Section of Agronomy and Field Crops, Department of Agriculture, Food and Environment, University of Catania, 95123 Catania, Italy; antonio.barbera@unict.it

**Keywords:** vitamins, phytochemicals, neurodevelopment assessments, maternal health outcomes, microbiota

## Abstract

Gestational diet has a long-dated effect not only on the disease risk in offspring but also on the occurrence of future neurological diseases. During ontogeny, changes in the epigenetic state that shape morphological and functional differentiation of several brain areas can affect embryonic fetal development. Many epigenetic mechanisms such as DNA methylation and hydroxymethylation, histone modifications, chromatin remodeling, and non-coding RNAs control brain gene expression, both in the course of neurodevelopment and in adult brain cognitive functions. Epigenetic alterations have been linked to neuro-evolutionary disorders with intellectual disability, plasticity, and memory and synaptic learning disorders. Epigenetic processes act specifically, affecting different regions based on the accessibility of chromatin and cell-specific states, facilitating the establishment of lost balance. Recent insights have underscored the interplay between epigenetic enzymes active during embryonic development and the presence of bioactive compounds, such as vitamins and polyphenols. The fruit of *Manilkara zapota* contains a rich array of these bioactive compounds, which are renowned for their beneficial properties for health. In this review, we delve into the action of each bioactive micronutrient found in *Manilkara zapota*, elucidating their roles in those epigenetic mechanisms crucial for neuronal development and programming. Through a comprehensive understanding of these interactions, we aim to shed light on potential avenues for harnessing dietary interventions to promote optimal neurodevelopment and mitigate the risk of neurological disorders.

## 1. Introduction

Neurodevelopment is a finely orchestrated process starting from gestation that continues until adulthood [1]. For the correct formation of the nervous system, the prenatal phase is extremely sensitive to lifestyle habits and environmental stimuli because of dynamic neurobiological events that occur during gestation. The maternal diet significantly influences the development of a healthy brain in the growing fetus, exerting a profound impact on its future functionality [2]. Variations in nutrition have potentially long-period consequences on offspring health and tendency to disorders later in life [3]. The epigenome begins to be encoded in the uterus and, during this period, the developing fetus is extremely vulnerable to nutritional status. During ontogenesis, variations in epigenetic status impact differentiation of precursor cells into their mature state [4], affecting embryo–fetal growth, and shaping the morphological and functional differentiation of the various brain regions. Additionally, it organizes embryo–fetal tissues and organs in response to the changing extra-uterine milieu by the modulation of metabolic–immuno–neuro–endocrine pathways [5]. Epigenetic machinery connects genes, the environment, and susceptibility to diseases. It embraces all those heritable modifications such as histone tail modifications, DNA methylation, ATP-dependent chromatin remodeling, and non-coding RNAs that cause variations in gene expression without altering DNA coding sequences [6], in this manner influencing gene transcription arrangements via various mechanisms and by controlling the reachability of genomic loci to numerous regulatory factors such as enhancers, silencers, and transcription factors [4]. Nowadays, there is an extraordinary focus on identifying available periconceptional nutrients that can affect the epigenetic state of the offspring and understanding how they can impact phenotypically. The critical window of vulnerability spans the first 1000 days from conception to the end of age 2, a period during which epigenetic mechanisms can significantly influence neurodevelopmental stages, namely neurogenesis, cell migration, differentiation, and synaptogenesis [7]. Neurulation starts in embryonic life, occurring on day 18 after conception and continuing until day 28 [3]. The sequence of cellular processes includes neurogenesis at day 42 to mid-gestation, followed by migration, differentiation, synapse formation, and apoptosis [8]. In the course of neurodevelopment, the epigenetic program organizes the development of the embryonic structures of the central nervous system, neurogenesis, and neuroplasticity. Altered epigenetic programming has been involved in the etiology of numerous neurological development conditions. Neurodevelopment is a particularly vulnerable phase to specific nutritional deficiencies [9]. Therefore, malnutrition in the early stages of life can favor dramatic and long-lasting alterations in development, leading to neurological diseases [10]. Nutritional deficits can be involved in molecular and cellular dysfunction, which trigger oxidative stress and inflammation, and can impact neuronal death, synapse and myelin formation, neurotransmitter metabolism, cell differentiation, plasticity, astrocytes, and axonal and dendritic growth. Overall, these events culminate in motor deficit and learning difficulties caused by delayed development of cognitive and motor skills [2]. Consequently, introducing important bioactive compounds early in development could be a determining factor in facilitating optimal neural plasticity during brain development and improving clinical outcomes [2]. Such compounds could influence the development and function of the brain and be involved in the fetal–neonatal disturbances predisposing to neurological delay or mental syndromes including autism spectrum disorder (ASD), attention-deficit/hyperactivity disorder, schizophrenia, epilepsy, anxiety, depression, cognitive dysfunction, visual impairment, motor deficits, and neural tube defects, such as spina bifida or anencephaly [11,12]. In recent years, it has been discovered that several epigenetic enzymes active during embryonic development require the presence of cofactors such as vitamins and polyphenols for their activity. Moreover, given the increasing incidence of neurodegenerative disorders, it is imperative to achieve significant advancements in the treatment of neurodegeneration by adopting different approaches. This includes the identification of nutrients that play an important role in either contributing to or being essential for the reduction of neurodegenerative disorder development.

**Figure 1:**
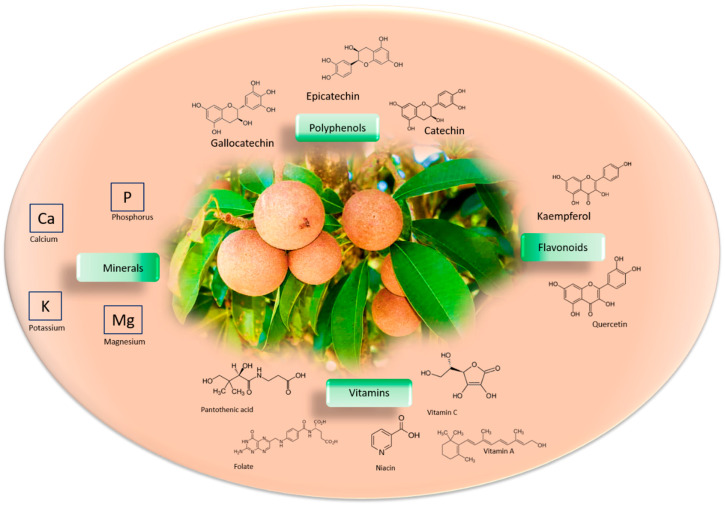
Phytochemical profile of *Manilkara zapota* fruit. Main structural formulas of bioactive compounds in the *Manilkara zapota*, like polyphenols, vitamins, minerals, and flavonoids.

One promising avenue involves investigating the potential of natural sources, such as the *Manilkara zapota* fruit, also known as “chicozapote”. This fruit is rich in bioactive compounds with a wide range of biological functions, such as antioxidant, antidiabetic, antimicrobial, anti-inflammatory, and cytotoxic agents. The beneficial properties of this fruit help prevent chronic and degenerative diseases, including cancer and diabetes, as well as neurological, infectious, and cardiovascular diseases. *Manilkara zapota* is an evergreen tree native to the regions of Central America (Mexico, Belize, and Guatemala) and Southeast Asia (Figure 1). The wealth of micronutrients presents in this fruit led us to investigate the complexities arising from the supplementation of micronutrients derived from this fruit on neurodevelopment. In this context, we explored the antioxidant effects of each of its biological compounds to understand, based on the findings from various studies, if the deprivation or integration of such micronutrients has an impact on the epigenetics of neurodevelopment. This review holds promise in shedding light on strategies for promoting healthy neurodevelopment and potentially mitigating the risk of neurodegenerative disorders through dietary interventions.

## 2. Micronutrients of *Manilkara zapota*: As Essential as Complex

*Manilkara zapota* fruit is a rich source of micronutrients such as minerals and vitamins, including vitamin A, B complex, C, folate, niacin, and pantothenic acid [13]. The mineral content of 100 g of *Manilkara zapota* fruit has been analyzed, and consists of 193 mg of potassium, 21 mg of calcium, 12 mg of magnesium, and same amount for phosphorus. *Manilkara zapota* phytochemical compounds are principally polyphenols, flavonoids, and tannins [14], which consist of ellagitannins and gallotannins [15] (Figure 1). The nutritional value of 100 g of *Manilkara zapota* fruit consists of 83 calories, 5.3 g total dietary fiber, 0.4 g protein, 1.1 g fat, and 20 g carbohydrates. Terpenes, alkaloids, saponins, steroids, and glycosides are also present [16]. The great calorie value is because of its rich carbohydrate level [17]. *Manilkara zapota* contains nearly 24 polyphenolic antioxidant compounds comprising methyl chlorogenate, myricitrin, (+)-catechin, (−)-epicatechin, (+)-gallocatechin, kaempferol, and dihydromyricetin [18,19] Methyl-4-*O*-galloylcholorogenate and 4-*O*-galloylchlorogenic acid have high antioxidant capacity [20]. *Manilkara zapota* has a total phenolic content (TPC) of 99.00 ± 12.30 mg of gallic acid equivalent (GAE)/(100 g) in fresh pulp [21], whereas TPC and total flavonoid content (TFC) in the peel is 1151.40 ± 32.3 GAE/(100 g) and 564.50 ± 30.50 quercetin equivalent (QE)/(100 g), respectively [22]. Therefore, *Manilkara zapota* is an excellent source of antioxidants. Using the 2,2′-azino-bis (3-ethylbenzthiazoline-6-sulfonic acid) (ABTS) assay, it has been reported that chicozapote fruit has an ascorbic acid equivalent antioxidant capacity (AEAC) of 3396 ± 387.9 mg/kg [23]. According to the 2,2-diphenyl-1-picrylhydrazyl (DPPH) assay, the elements with the highest degree of antioxidant capacity isolated from methanolic fruit extract were methyl-4-*O*-galloylchrologenate and 4-*O*-galloylchlorogenic acid [24]. Fresh pulp extract showed a DPPH potential that was more robust than dried fruit, implying that the drying procedure can influence antioxidant potential [25]. The shelf life of the fruits at 12–16 °C is reported to be 3 weeks at 85–90% relative humidity [26].

Various food products from *Manilkara zapota* such as jam, gelatins, chutney, dehydrated slices, candy, syrup, and mixed *Manilkara zapota* juices can be created to manage the perishability and preserve the wealth of its bioactive compounds [27].

## 3. Bioactive Compounds Deficiency: Still Lacking the Crucial Knowledges

Bioactive compound deficiency may affect the maternal immune response and metabolic decompensation, which are essential factors responsible for epigenetic dysregulation [28]. In cooperation, the maternal immune response and epigenetic mechanism control ontogenesis and neurodevelopment. Maternal immune activation affects the remodeling of some chromosomal areas, transforming gene expression in the brain of offspring and generating changes with long-term effects [28]. Maternal metabolic imbalance also triggers strong cellular alterations, with variations at the molecular level. Supplementation of biologically active compounds such as polyphenolic substances, vitamins, and minerals can influence molecular/cellular processes, reducing oxidative stress, epigenetic modifications, and alterations in lysosomal activity modulating gene expression or protein content, which preserves neurodevelopment [29]. In the same way as developmental neurogenesis, adult neurogenesis is controlled by extrinsic and intrinsic influences such as micronutrients influencing epigenetic phenomena [30]. In adults, micronutrient shortage can promote several neurological illnesses such as depression, confusion, dementia, optic neuropathy, and numbness and tingling in limbs [31]. Additionally, bioactive compound deficiency is linked with cognitive disorders. Although not fully confirmed, some researchers assume that bioactive compound deficiency can constitute an opportunity for the development of several neurological disorders such as Alzheimer’s disease (AD), Wernicke’s encephalopathy, and subacute combined degeneration of the spinal cord and peripheral neuropathy [31].

## 4. Bioactive Compounds Are Valuable in Epigenetic Machinery

**Figure 2:**
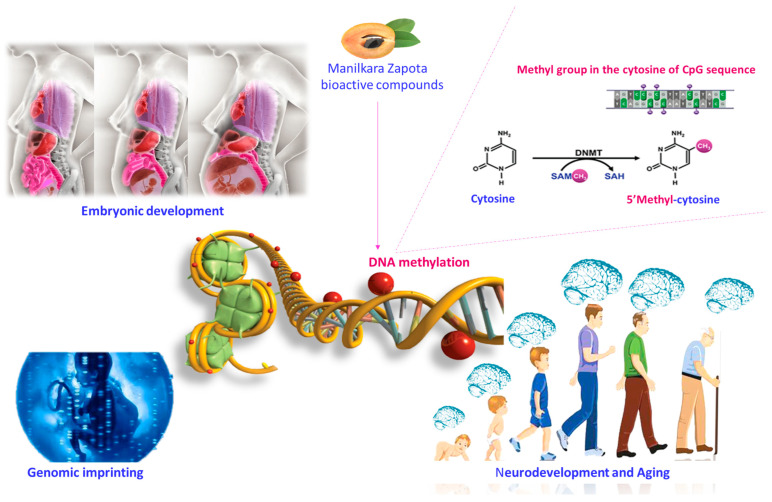
Potential influence of bioactive compounds of the *Manilkara zapota* fruit on DNA methylation. Bioactive compounds of the *Manilkara zapota* fruit contribute to the production of SAM and are linked to epigenetic mechanisms, favoring DNA methylation. Supplementation during pregnancy and lactation can influence embryonic development, genomic imprinting, and aging. Abbreviations: SAM (S-adenosyl-L-methionine); SAH S-adenosylhomocysteine.

Bioactive compounds regulate the epigenetic remodeling of fetal genes. Their beneficial effects are evident from the implant, affecting placental development and nutrient transfer [32]. Bioactive compounds control the biological functions that influence neurodevelopment modifying DNA methylation configurations [33]. DNA methylation is a reversible and active process mainly responsible for long-lasting gene silencing. Furthermore, DNA methylation allows the gene expression regulation, affecting various biological functions comprising embryonic growth, genomic imprinting, and aging. DNA methylation takes place when a methyl group is positioned on the cytosine of a phosphodiester-bonded cytosine–guanine dinucleotide (CpG) sequence. The covalent addition of a methyl group from S-adenosyl-l-methionine (SAM), to the C-5 position of cytosine bases occurs via the action of a group of DNA methyltransferase enzymes (DNMTs) [34]. SAM is produced by one-carbon metabolism, including the folate and methionine cycles, embracing the transformation of 5,10-methylenetetrahydrofolate to 5-methyltetrahydrofolate, which is catalyzed by methylenetetrahydrofolate reductase (MTHFR). In the context of DNA methylation, DNMTs function as “writers”, whereas methyl-CpG-binding domain proteins (MECPs), which recognize methylated CpGs, function as “readers” [35]. Some bioactive compounds of *Manilkara zapota*, such as folate, choline, betaine, methionine, and vitamin B_6_, participate in one-carbon metabolism, contributing to SAM production, and are linked with DNA methylation (Figure 2) [36]. Other compounds, such as epigallocatechin-3-gallate (EGCG) and quercetin, are potent inhibitors of DNMTs. They act by binding the active site of the enzymes and, thus, reducing DNA methylation [37].

## 5. Role of Vitamin A in Brain Epigenetic Phenomena

**Figure 3:**
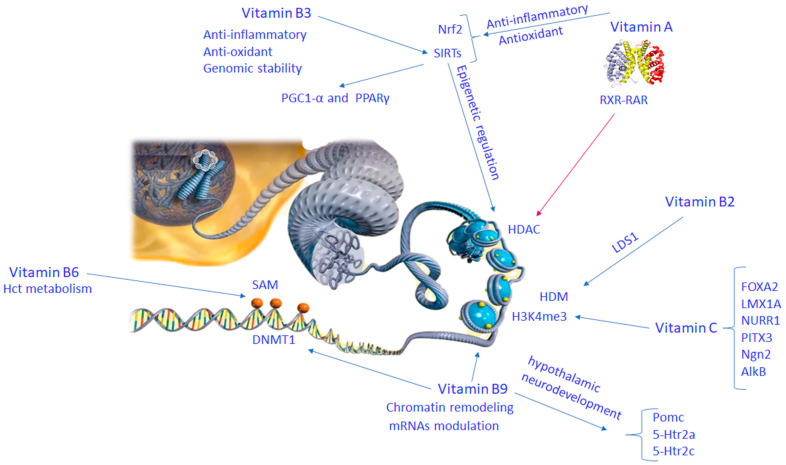
Molecular mechanisms of vitamin B complex and vitamin C involved in brain epigenetic phenomena. Abbreviation: 5-HTR2C (5-hydroxytryptamine receptor 2C); ALKB (alkylation repair enzyme AlkB homolog 1); FOXA2 (forkhead box protein A2); H3K4ME (histone H3 lysine 4 methylation); HCT metabolism (histidine catabolism metabolism); HDAC (histone deacetylase); HDM (histone demethylase); 5-HTR2A (5-hydroxytryptamine receptor 2A); LDS1 (lysine demethylase 1); LMX1A (LIM homeobox protein 1A); NGN2 (neurogenin 2); NRF2 (nuclear factor (erythroid-derived 2)-like 2); NURR1 (nuclear receptor related 1); PGC1A (peroxisome proliferator–activated receptor gamma coactivator 1 alpha); PITX3 (paired homeobox protein 3); PPAR-γ (peroxisome proliferator–activated receptor gamma); RXR-RAR (retinoid X receptor–retinoic acid receptor); SAM (S-adenosylmethionine); SIRTS (sirtuin family of proteins).

Vitamin A is transported in the blood as retinol. Its metabolite, retinoic acid (RA), is required in various processes in different tissues. RA is crucial for brain and neural system development starting from the embryonic stage [38]. In neurons, RA regulates differentiation, tissue formation, and synaptic plasticity through its binding with retinoic acid receptors (RARs), sited in the promoter regions of RA-responsive genes, and affecting the binding of other transcription factors [39]. RA molecular signaling pathways involved in neuronal differentiation are intricate. In particular, through the homeobox (*Hox*) gene clusters control several developmentally important genes [40]. Following RA stimuli, heterodimers of RARγ and retinoid X receptor α (RXRα) recognize and bind RA-responsive DNA elements (RAREs), thereby affecting epigenetic variations on histones, generating heritable alterations in chromatin responsiveness (Figure 3) [41]. Vitamin A deficiency (VAD) is a public health concern, especially in some developing countries. VAD is found in about 19 million pregnant women and 190 million children worldwide [42]. During the embryo or postnatal period, VAD generates disorders of the RA signaling pathway. VAD-related genetic and molecular defects linked with low serum levels of RA, beta-carotene, and retinal dehydrogenase 1 have been found in children with ASD [43]. During pregnancy, low VA serum levels in ADS children are often accompanied by multivitamin deficiency and by atypical eating patterns such as pickiness, food refusal, and diet restriction, which may further exacerbate nutritional deficiencies [44]. Stem cell treatment with RA modulates many enzymes that modify the histones engaged in the transcriptional activation of certain genes. In particular RA, by binding to the RARs, modifies connections of the RARs with proteins belonging to the transcription complex on numerous genes in stem cells. Some of these elements of the transcriptional complex insert or remove epigenetic marks on histones, modifying the chromatin structure and inducing the exodus from the self-renewing pluripotent stem cell condition [45]. It has been found that crucial gene expression modifications, facilitated by miRNAs, support the regulation of embryonic stem cell (ESC) RA-associated differentiation [45]. It has been suggested that histone demethylases expedite DNA demethylation in the promoter region of miR-219, thus promoting miR-219 expression and neural differentiation. High expression levels of miRNA-219 may be critical during the early stage of RA-induced neural directional mESC differentiation [46]. During neural differentiation, Foxj3 and Zbtb18, which are two target genes of miR-219, are known to be responsible for inducing oligodendrocyte differentiation and in part rescuing oligodendrocyte differentiation failure produced by total miRNA defeat. However, *Olig1* and *Olig2* are also crucial components in the neural differentiation of ESCs. *Olig1*/*2*-knockdown ESCs, cultured in the presence of RA or transfected with miR-219 mimic, were unable to differentiate into neural cells. Particularly, nestin (neural stem cell marker) was raised in the *Olig1*/*2*-knockdown ESCs treated with RA or miR-219 mimic.

The gradation of acetylation histones and transcription factors influences neuronal differentiation [47]. It has recently been found that RA treatment modulates SIRT1 expression [48].

SIRT1 catalyzes the deacetylation reaction on both non-histone and histone proteins that regulate metabolic responses to stress signals [49]. SIRT1 reduces inflammatory responses and oxidative stress, preventing the onset of neurological diseases [50].

SIRT1 absence delays axogenesis and dendritic splitting in embryonic hippocampal neurons [48]. Therefore, SIRT1 is important, starting from the early phases of neuronal differentiation. SIRT1 behaves as a co-repressor of nucleolar RAR, and its shortage increases the acetylation condition of cellular retinoic acid binding protein II (CRABPII), stimulating differentiation of murine cells ESC through RA signaling [48]. In adulthood, VAD is engaged in the pathogenesis and evolution of AD. The increased expression of oxidative stress and neuroinflammatory genes is consistent with all-*trans* retinoic acid (ATRA) depletion. ATRA is a metabolite of VA in the brain. It has different functions in the human hippocampus, as demonstrated by the finding that several genes mediating retinol transport, ATRA synthesis, and ATRA metabolism are compromised in hippocampal tissue from post-mortem AD brains [51]. Activation of NFKB1 and NFKB2 is linked to neuroinflammation following oxidative stress. Interference between Nrf2- and RAR-mediated signaling reduction by the action of VA has been reported, suggesting that VA can act as an antioxidant. ATRA deficiency can be related to age-dependent epigenetic silencing [52]. It has been found that an enhanced expression of HDACs and abnormal epigenetic silencing induces the downregulation of RAR- and Nrf2-dependent genes in AD. Therefore, VA supplementation inhibiting HDAC inhibition could avoid AD (Figure 3) [53].

## 6. Role of Vitamin B Complex in Brain Epigenetic Phenomena

During pregnancy, exposure to water-soluble complex B vitamins influences the epigenetic signature in neurodevelopment. These effects are linked with varied gene expressions associated to brain development, axon guidance, and oxidative stress in the hypothalamus of newborns. Complex B vitamins comprising B2 and B3 exert anti-inflammatory effects, modulating genes such as NF-κB activation, IL1B, IL6, IL10, iNOS, COX2, NFκB, GSK-3β, TNF, APP, and several miRNAs stimulated by diacetyl benzene (DAB) in human neuroblastoma SH-SY5Y cells [54]. DAB is a molecule that passes through the blood–brain barrier (BBB), producing neuroinflammation, tau hyperphosphorylation, and cognitive deficiency (Figure 3).

### 6.1. Vitamin B2

Vitamin B2 or riboflavin reduces cognitive impairment via antioxidative stress and anti-inflammation properties. This vitamin is engaged in the modification of epigenetic signatures. Vitamin B2 deficiency generates modifications in the methylation process. Vitamin B2 facilitates the one-carbon group metabolism essential for histone function and the S-adenosylmethionine reaction [55]. Vitamin B2 controls histone methylation by inducing lysine-specific demethylase 1 (LSD1), which causes deletion of methyl groups from the H3 element of histone (Figure 2). This epigenetic effect is facilitated by the action of LSD1 via redox reactions. Oxygen molecules lead to the oxidation of FADH2 into FAD. LSD1, removing mono and dimethyl groups by oxidative separation, reduces flavin in 1,5-dihydro flavin adenine dinucleotide (FADH2) [56].

### 6.2. Vitamin B3

Vitamin B3 or niacin plays an important role in the functioning of the nervous system, and is involved in cell differentiation and apoptosis. Vitamin B3 promotes numerous cellular pathways, such as cell signaling, redox reactions, activation of nucleotide syntheses, and folate-dependent enzymes [57], and facilitates transcriptional activity, recombination, DNA repair, and genome stability [58]. Vitamin B3 deficiency leads to DNA fragility and damage [57]. Therefore, niacin supplementation mitigates genomic instability [59]. Vitamin B3 is deemed as a cofactor of NAD^+^-dependent enzymes, including PARPs, SIRTs, and ADP-ribosyl transferase. Both calorie control and oxidative stress impact the action of these enzymes by varying the NAD^+^/nicotinamide adenine dinucleotide +hydrogen (NADH) ratio [60]. SIRTs are NAD^+^-dependent enzymes involved in deacetylation and ADP-ribosyltransferase activities required by transcription factors, co-regulation factors, histones, and enzymatic reactions. The SIRT family includes SIRT1, SIRT6, and SIRT7. They preserve genome integrity, supporting DNA repair and genome configuration, whereas SIRT2 contributes to cell cycle regulation [61]. SIRT1 function is regulated by nicotinamide adenine dinucleotide (NAD^+^) and it is consequently sensitive to the redox condition and cellular metabolism. SIRT1 activation has been shown to be related to a shift into the astroglial lineage in neural progenitor cells (NPCs) [62]. A reduction in NAD levels may be associated with reduced SIRT1 activation in promoting neuron differentiation. In addition, Sirt1-deficient mice display severe neural failings such as interrupted neuroretinal morphogenesis and exencephaly [63]. SIRTs cooperate with PGC1-α and PPARγ as key transcription factors (Figure 3). The activities attributed to PARPs include poly ADP ribose sequence generation [64], protection against chronic genotoxic stress, protection of chromosome endpoints to preserve genome integrity, and telomerase regulation. SIRT proteins control epigenetic pathways modulating histone acetylation and chromatin-associated enzyme activities.

### 6.3. Vitamin B6

Vitamin B6 is one of the critical factors for homocysteine (Hct) metabolism. Hct is an important amino acid available from a daily diet. It is produced in the metabolism of methionine. Hyperhomocysteinemia (HHct) is triggered by several factors, such as genetic deficits, and vitamin B6 and folic acid deficiency. A recessively inherited defect producing a deficiency of the enzyme 5,10-methylenetetrahydrofolate reductase (MTHFR) is the origin of one form of hyperhomocysteinemia. Severe MTHFR deficiency generates neurological manifestations, in particular neurodevelopment abnormalities and evolution of neuropsychiatric disorders such as ASD, schizophrenia, and epileptic seizures. Taking into account the important role of vitamin B6 as a cofactor of enzymes involved in SAM generation, its deficiency impacts the epigenetic configuration of the genome via the methylation pathway [65] (Figure 3). In addition to DNA hypomethylation, vitamin B6 deficiency, due to decreased levels of thymidine in the DNA, causes increased chromosomal breakdown and impairment of DNA excision repair [66]. This evidence indicates that methyl donor availability can influence epigenetic modifications and oligodendrocyte differentiation [67].

### 6.4. Vitamin B9

Folate is involved in the one-carbon group’s metabolism and methionine synthesis [68]. Its primary function lies in cell division activities, such as synthesis, expression, and DNA repair [69]. 5,10-methylenetetrahydrofolate (*N*-5,10 mTHF) is the metabolically active form of folate formed by the transformation of serine into glycine in the course of the insertion of a methylene group from tetrahydrofolate. Acting as a crucial coenzyme, *N*-5,10 mTHF governs the synthesis of DNA precursors, serving as a rate-limiting factor [57]. Additionally, it supplies methyl donors for all methyltransferase enzymes such as hydroxy indole-*O*-methyltransferase, catechol-*O*-methyltransferase, and phenylethanolamine-*N*-methyltransferase [69]. The bioactive metabolite 5-methyltetrahydrofolate (5-MTHF, L-methylfolate) stabilizes the synthesis of the monoamine neurotransmitters dopamine, norepinephrine, and serotonin via the tetrahydrobiopterin pathway [70]. Folate is involved in chromatin remodeling, in micro-RNAs, and post-translational modification of histone proteins [71]. The methylation of CpG-dinucleotides is essential in the regulation of transcription pathways and cell division, growth, and differentiation [72].

Folate deficiency causes DNA hypomethylation, in all regions of DNA breakage and inactivated repair [73], and in specific genes involved in genomic stability affecting the occurrence of severe neurological abnormalities such as tumors and cerebrovascular diseases [74]. In the course of brain development, folate is required for neural stem cell (NSC) proliferation and differentiation. Studies on primary embryonic cells isolated from rat fetuses indicate that maternal folic acid deficiency reduces cell proliferation, impairs mitosis, and increases apoptosis [75]. Notably, folate deficiency is not only the main cause of neural tube defects but it has also been found in several congenital defects including cognitive dysfunction and ASD, and also in dementia and AD [76]. Experimental evidence suggests that folate deficiency can induce neuronal immaturity via DNA methylation. Primary mouse NSCs/NPCs in a low-folate medium exhibited reduced SAM levels and DNA hypomethylation in the CpG islands within the promoters of genes crucial for neuronal functions, including *Neurog1*, *Eomes*, and *Neurod1* [77]. Another study showed that, during gestation, folic acid deficiency reduces NSC proliferation and increases apoptosis in the fetal brain [78]. Maternal folic acid deficiency further damages NSC differentiation and induces apoptosis of neurons in the fetal brain via *ERK1/2* gene pathways [79]. During pregnancy and lactation, folic acid deficiency induced plasma peptide YY and POMC gene expression in rat offspring, leading to anomalous leptin, ghrelin, and insulin secretions, affecting hypothalamic neurodevelopment [80]. Moreover, post-weaning dietary folic acid content affects hypothalamic neurodevelopment through epigenetic mechanisms attenuating the decrease in Pomc mRNA expression and gene POMC promoter DNA methylation levels [77] (Figure 3). A large amount of folic acid (10 times more than recommended) in the offspring diet changes *5-Htr2a*, *5-Htr2c,* and *Pomc* hypothalamic gene expression [77].

This suggests that neurodevelopment plasticity of hypothalamic neuro-circuits in the course of the pre- and postnatal stages is susceptible to changes in the content of vitamins in the diet.

## 7. Vitamin C in Brain Epigenetic Phenomena

Vitamin C is an important bioactive molecule in humans. Vitamin C is a cofactor of the demethylation of histone and DNA breakdown enzymes essential in the setting of epigenetic phenomena. Vitamin C in DNAm operates through the activation of TET hydroxylase enzymes [81]. The maximum quantity of vitamin C has been observed in the brain, mainly in the course of embryonic development [82]. Vitamin C deficiency causes disorders of epigenetic patterns and incidence of neurodegenerative disorders [82]. Vitamin C enhances upregulation of FOXA2 and LIM homeobox transcription factor 1 alpha (LMX1A) markers in the course of differentiation of neurons by increasing expression of the associated genes, such as nuclear receptor-related 1 protein (NURR1), pituitary homeobox 3 (PITX3), and neurogenin 2 (Ngn2) [83], and recovers defects in neurons due to degenerative brain diseases [82]. Without a doubt, vitamin C induces mRNA expression of these genes during the transcription phase. Vitamin C is also a cofactor of Fe2^+^-oxoglutarate-dependent dioxygenase enzymes (ten–eleven-translocation) and jumonji (JmjC)-domain-containing enzymes in the demethylation of lysine residues of histone proteins [84]. In adequate doses, vitamin C can preserve the action of histone H3 acetylation and Lys4 methylation (H3K4me3) [85] (Figure 3). A substantial correlation has been found between upregulation of TET enzymes and high levels of 5-hydroxymethylcytosine in CpG sites of the promoter region [83]. It has been shown that TET1 and TET2 activities are displayed in DNAm patterns and enhance reprogramming capability [86]. In addition, vitamin C activates the AlkB family protein, which mediates nucleic acid repair [86]. In particular, AlkB and its mammalian homologs, ABH2 and ABH3, facilitate DNA repair by oxidation of 3-methylcytosine and 1-methyladenine [87].

## 8. The Neuroprotective Effects of Polyphenols: A Story Told by Neuroinflammatory Disorders

Polyphenols are heterogeneous compounds with strong antioxidant properties broadly distributed in plants in the form of glycosides or aglycones. Chemically, they are molecules typified by at least two phenol rings and one or more hydroxyl substituents. Polyphenols can be distinguished into many subclasses depending on the amount of phenolic units contained in their molecular organization. For the substituent groups and/or the nature of bond between phenolic rings, they can be classified into flavonoids and non-flavonoids. The principal subclasses of polyphenols embrace phenolic acids, coumarins, stilbenes, flavonoids, tannins, and lignans [88]. It has been reported that polyphenol supplementation during pregnancy and breastfeeding may moderate brain injury in embryonic, fetal, and neonatal subjects, as well as offspring, emphasizing the role of these organic substances in controlling adaptative responses involving phenotypical plasticity. The brain is predisposed to oxidative stress because of its elevated oxygen intake, low antioxidant aptitude, and high levels of polyunsaturated fatty acids which are susceptible to lipid peroxidation [89]. Polyphenols restrain inflammation and oxidative stress that origin permanent damages in movement, cognitive, and behavioral functions, such as cerebral palsy, hydrocephaly, blindness, and deafness [90].

**Figure 4:**
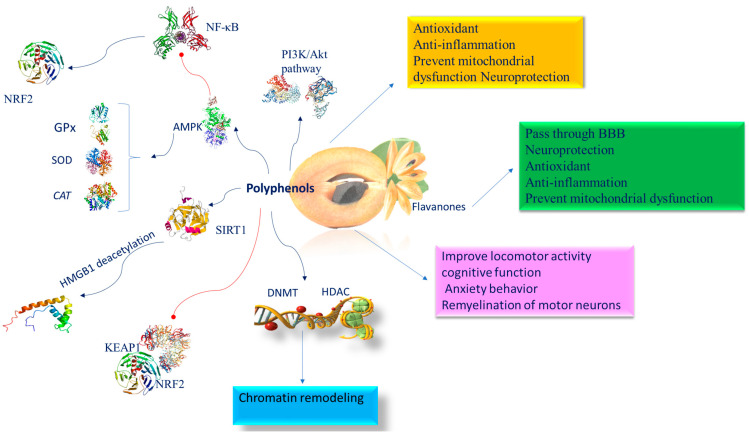
Molecular mechanism of polyphenols involved in brain epigenetic phenomena. Abbreviation: AMPK (AMP-activated protein kinase); BBB (blood–brain barrier); CAT (catalase); DNMT (DNA methyltransferase); GPx (glutathione peroxidase); HDAC (histone deacetylase); NF-κB (nuclear factor kappa-light-chain-enhancer of activated B cells); KEAP1 (Kelch-like ECH-associated protein 1); NRF2 (nuclear factor (erythroid-derived 2)-like 2); PI3K/AKT (phosphatidylinositol 3-kinase/AKT); SIRT1 (sirtuin 1); SOD (superoxide dismutase).

One of the most significant roles of phenols is their capacity to prevent free radical formation. They operate as metal chelators, free-radical scavengers, reducing agents, enzyme regulators of various proteins, and transcriptional factors [91]. The antioxidant propriety of these molecules confers neuroprotective effects, thus delaying the onset or decreasing the risk of neurodegenerative diseases, such as AD, Parkinson’s (PD), dementia with Lewy bodies, and multiple system atrophy [92]. Their bioactivity is also attributed to their capacity to inhibit the GABA receptor [93], prevent mitochondrial dysfunction [94], and control neuronal signaling pathways essential for monitoring neuronal resistance to neurotoxic oxidants, inflammatory mediators, and chelation of transition metal ions [94]. The placenta, regulating the transition of nutrients between maternal and fetal blood, represents a symbiosis point between the mother and the fetus. The placenta is the first fetal line of defense in the course of pregnancy. Furthermore, when the organism is particularly exposed to external stimuli, physiological barriers such as the BBB, which controls selective permeability, are crucial for healthy neurodevelopment. The BBB begins to be operational around the eighth week of gestation, while, at the twelfth week, tight junction proteins are expressed [95]. The tight junctions providing semi-permeability act as an obstacle to the free entrance of molecules from the early stages of brain development [95]. Polyphenols, like some flavanones, can pass through the BBB and directly protect neurons because of their antioxidant properties and their direct effects on enzymes, proteins, receptors, and signaling pathways. Epigenetic modifications could activate genes related to the phosphatidylinositol 3-kinase (PI3K)/protein kinase B (Akt) signaling pathway. Histone and DNA methyltransferase changes activate AMP-activated protein kinase (AMPK) as a strategic actor in epigenetic regulation. The remarkable efficiency of polyphenols is related to a number of signaling pathways involving AMPK, nuclear factor kappa B (NF-κB), and PI3K. For instance, lack of oxygen is frequently linked with reduced AMPK expression, which may be induced by polyphenol supplementation, which increases levels of the antioxidant system’s SOD, glutathione peroxidase, and catalase, and decreases the expression of inflammatory mediators through NF-κB suppression [96]. Treatment with polyphenols during pregnancy [97] and lactation [98] improves locomotor activity, cognitive function, and anxiety behavior in offspring exposed to brain damage. Moreover, they can reduce free radical formation and neuroinflammation in the hippocampus [99], and ameliorate remyelination of motor neurons [100]. The neuroprotective activity exerted by polyphenols depends as well as on their antioxidant and anti-inflammatory ability [101], and on their antimutagenic and antimicrobial activity in the activation of enzymes responsible for antioxidant systems and xenobiotic detoxification [102]. Inflammation is linked with increased DNA methylation levels, [103], which disturb the expression of genes engaged in the crosstalk between inflammatory pathways and neural activity, finally providing susceptibility to psychopathologies, including depression and anxiety [104]. Oxidative stress has a double effect: on the one hand, it deactivates histone deacetylase 2 (HDAC2), and, at the same time, it activates the DNA methylating enzyme, DNMT1 [105]. Polyphenols influence epigenetic alterations such as chromatin remodeling through modulation of histone deacetylase (HDAC) and DNA methyltransferase (DNMT) actions, which, therefore, can invert atypical gene expression [105]. Polyphenols trigger SIRT1 to deacetylate high-mobility group box-1 (HMGB1) thus reducing its translocation and preventing the inflammatory response induced by the toll-like receptor 4 (TLR4) signaling pathway [106]. In addition, polyphenols protecting SIRT1 against oxidative stress effects prevent neurodegeneration and cognitive impairment [107]. Polyphenol antioxidant properties are dependent on the inhibition of NO and PGE2 release, and the consequent activation of NADPH oxidase and prevention of ROS formation in the brain. Furthermore, interacting with and activating the receptor kinases ERK1/2, JNK, and p38, they interfere the Keap1/Nrf2 complex and induce the translocation of nuclear factor 2 (Nrf2)-related transcription factors to the nucleus, where, by binding to receptors rich in adenylate and uridylate (ARE), they promote the action of antioxidant proteins and enzymes [108]. Epigenetic modifications could activate genes linked to the phosphatidylinositol 3-kinase (PI3K)/protein kinase B (Akt) signaling pathway. It has been shown that, following insults, polyphenols restored the impaired P13K/Akt pathway, the dimensions of cerebral infarct and apoptosis interrupting caspase-3 activation, together with a decline in Bcl-Xl and Bcl-2 expression and proteolytic processing of post-lesion substrates [109,110] (Figure 4).

### Epigallocatechin Gallate (EGCG)

Among the polyphenolic antioxidants isolated from *Manilkara zapota*, epicatechin gallocatechin, because of its multiple biological effects, is one of the most studied catechins. It crosses the BBB, which is a prerequisite for the effectiveness of epigallocatechin-3-gallate (EGCG) against neurodegenerative disorders [19]. The antioxidant action of EGCG has been observed in neuroprotective prenatal treatments in the course of intrauterine growth restriction (IUGR) [111], which is a common disease throughout development. IUGR is designated as a substantial decrease in the rate of fetal development, principally consequential to placental insufficiency [111]. This occurrence entails a fetal development in conditions of chronic hypoxia, producing lesions of the white substance as a result of myelination defects and an important decrease in oligodendrogenesis [111]. In the long term, IUGR can result in serious outcomes of neurodevelopment such as neurocognitive disorders, learning difficulties, attention deficit disorder or hyperactivity, and autism spectrum disorder [111]. EGCG exerts neuroprotective properties by recovering cerebral functions and reducing ROS generation. In the middle cerebral artery occlusion rat model of cerebral I/R injury, it has been found that EGCG promotes NRF2 translocation into the nucleus and triggers the NRF2/ARE pathway and its downstream target genes, such as the glutamate–cysteine ligase modulatory subunit and glutamate–cysteine ligase regulatory subunit [112]. Among its properties, EGCG regulates several enzymes by modulating their kinase activity [113]. EGCG, in some measure, preserves the hippocampus from the effects of overexpression of dual specificity tyrosine-phosphorylation-regulated kinase 1A (DYRK1A), which is engaged in neurodevelopment. EGCG inhibition of *DYRK1A* kinase activity causes stabilization of this gene, which is crucial for Down syndrome pathology [114]. In addition, DYRK1A is engaged in epigenetic regulation, suggesting that EGCG could both directly and indirectly control the epigenetic state. This association is very vigorous since, like the EGCG effect, an enriched milieus repairs defects in Down syndrome, stabilizes DYRK1A levels, and interferes at various levels with epigenetic mechanisms [115]. EGCG affects the chromatin state and prevents both DNA methyltransferases [116], HAT [117], and class I histone deacetylases (HDAC 1, 2, 3, and 8) [118]. It influences the anti-NF-kB transactivation in a wide array of human chronic inflammation diseases. EGCG reduces ac-RelA (K310) acetylation by preventing the activity of HAT enzymes, p300, and CPB [117]. This hypoacetylation of p65 leads to the downregulation of NF-kB function by diverse inflammatory signals. EGCG inhibition of HAT can result in the failure of histone acetylation in HDAC promoter, preventing HDAC expression, thus contributing to its neuroprotective effect. EGCG decreases H327me3 and H2AK119 ubiquitination moderating polycomb protein levels [119] and miRNA expression [120], and thus improves cognitive and neural plasticity phenotypes [114] (Figure 4).

## 9. Phenolic Acids of *Manilkara zapota* Exerting Neuroprotective Functions

The most abundant phenolic compounds found in *Manilkara zapota* are gallic acid (GA) and protocatechuic acid, accompanied by chlorogenic, ferulic, caffeic, vanillic, and coumaric acids [17]. Improvements in cognitive functions due to these phenolic compounds have been observed in several neuropathological disorders, such as cerebral ischemia [121,122,123], LPS-induced neurological alterations [124], and scopolamine-induced neurotoxicity [125]. They share antioxidant, anti-inflammatory, neuroprotective [126], and memory-ameliorating properties [124].

### 9.1. Gallic Acid Epigenetic Mechanisms

GA is a 5,4,3-trihydroxybenzoic acid found copiously in free, conjugated (hydrolyzable tannins), and esterified forms [127]. GA prevents the nuclear translocation of p65 of NF-κB caused by lipopolysaccharide (LPS). Moreover, GA inhibits several histone acetyltransferases (HATs) [128], including HDAC and histone methyltransferase (HMT). GA can regulate key signaling pathways implicated in inflammatory responses such as the PI3K/AKT/mTOR pathway [129]. Evidence reported that GA inhibits p300-induced p65 acetylation and p300/CBP-dependent HAT activities [130].

**Figure 5:**
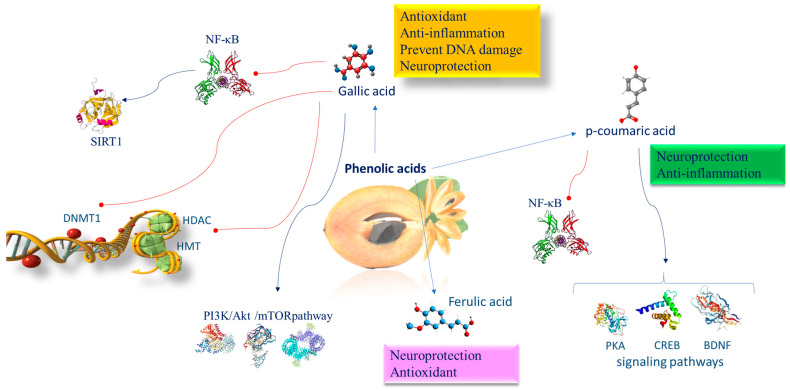
Molecular mechanism of phenolic acids involved in brain epigenetic phenomena. Abbreviations: BDNF (brain-derived neurotrophic factor); CREB (CAMP response element binding protein); DNMT1 (DNA methyltransferase 1); HDAC (histone deacetylase); HMT (histone methyltransferase); NF-κB (nuclear factor kappa-light-chain-enhancer of activated B cells); PI3K/AKT/mTOR (phosphatidylinositol 3-kinase/AKT/mammalian target of rapamycin); PKA (protein kinase A); SIRT1 (sirtuin 1).

GA displays a strong inhibitory role on DNMT1 activity, preventing Akt phosphorylation. In addition, it reduces both the nuclear import and protein stability of DNMT1. DNMT1 is known as an important enzyme working in the somatic inheritance of DNA methylation and therefore is indispensable in epigenome preservation [131]. GA induces SIRT1 to protect cells against oxidative stress [132]. It has been proven that SIRT1 deacetylates lysine 310 of the RelA/p65 subunit of NF-kB, a critical subunit for the activation of proinflammatory gene transcription that generates the inflammatory response [133]. In the brain, inflammation is largely linked to glia cells, which produce cytokines and influence synaptic plasticity in neurons [134]. NF-κB comprises the heterodimeric complex of p50/p52 and p65 proteins. In the cytoplasm, the NF-κB heterodimer links the inhibitory protein IκB, inactivating the whole complex. ROS and other proinflammatory factors induce protein kinase that phosphorylates IkB, which releases the p50/p65 complex. Then p50/p65 translocates to the nucleus where, by binding to specific DNA promoter regions, it functions as a transcription factor [135]. In particular, p65 is a subunit transcriptional activation domain of NF-κB and is controlled by posttranslational changes such as phosphorylation serines (276, 311, 529, and 536) and acetylation lysines (310, 122, 123, 218, and 221) [136]. Neuroprotection is linked with variations in the acetylation state of NF-κB/RelA. The acetylation of lysine 310 of the RelA/p65 NF-κB subunit protracts NF-κB activation time and increases its efficiency, amplifying inflammation [137]. The SIRT1 deacetylase enzyme can cooperate with the RelA/p65 protein complex NF-κB, deacetylating lysine 310 of the RelA/p65 NF-kB subunit to avoid transcription of proinflammatory genes [137] (Figure 5). It has been shown that GA, because of its antioxidant, anti-inflammatory, and anti-amyloidogenic effects, overcomes microglial-mediated neuroinflammation and reduces cytokine generation [138]. Moreover, the GA neuroprotective action has been demonstrated in experimental models of traumatic brain injury attributable to enhancement in the antioxidant and anti-inflammatory functions [139]. In multiple sclerosis experimental animals, GA treatments reduced the oxidative and inflammatory response and stimulated dendritic hyperplasia with an intensification of dendritic spines [140]. Structural and functional variations in the neurons of lab animals with neurodegenerative diseases were restored after GA administration, and, as a result, neurochemical functions were reestablished [141].

### 9.2. Epigenetic Mechanisms of p-Coumaric Acid

*p*-CA supplementation exerts beneficial effects in patients with stress-associated mental disorders, attenuating memory deficits and depression-like behavior. *p*-CA is engaged in numerous targets and a large quantity of signaling pathways, such as the protein kinase A (PKA)–cAMP-response element binding protein (CREB)–brain-derived neurotrophic factor (BDNF) pathway. Moreover, *p*-CA inhibiting NF-κB activation reduces neuroinflammation in a murine model with induced chronic unpredictable mild stress in the prefrontal cortex [142] (Figure 5).

### 9.3. Epigenetic Mechanisms of Ferulic Acid

FA prevents the decline in the diameter and thickness of hippocampal capillaries, so supporting oxygen and nutrient sources and the elimination of metabolic waste from the brain, which lastly leads to recovering spatial memory and also reduces memory loss (Figure 4). FA increases neuronal survival, enhances antioxidant enzyme function, modulates neuronal signal transduction, and impairs cholinesterase activity (ChAT) [143].

## 10. Flavonoids of *Manilkara zapota* Having Neuroprotective Functions

**Figure 6:**
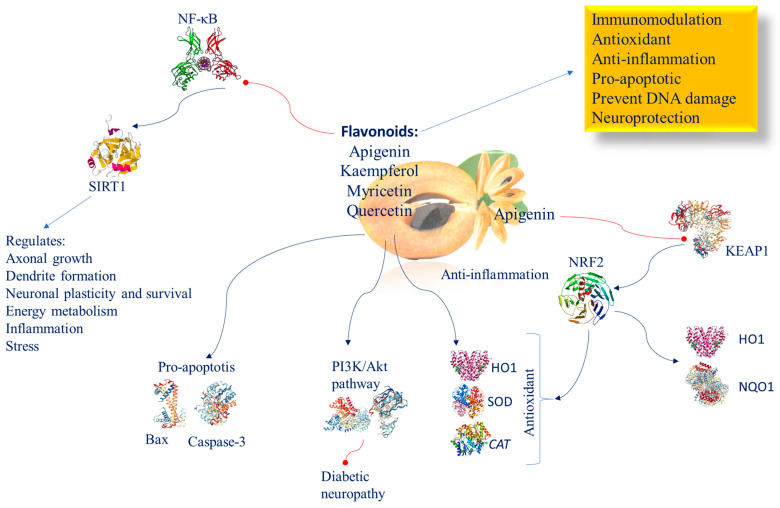
Molecular mechanism of flavonoids involved in brain epigenetic phenomena. Abbreviation: BAX (Bcl-2-associated X protein); CAT (catalase); HO1 (heme oxygenase 1); KEAP1 (Kelch-like ECH-associated protein 1); NF-κB (nuclear factor kappa-light-chain-enhancer of activated B cells); NQO1 (NAD(P)H:quinone oxidoreductase 1); NRF2 (nuclear factor (erythroid-derived 2)-like 2); PI3K/AKT (phosphatidylinositol 3-kinase/AKT); SIRT1 (sirtuin 1); SOD (superoxide dismutase).

Flavonoids present in *Manilkara zapota* include quercetin, apigenin, kaempferol, and myricetin. It has been demonstrated that different flavonoids induce SIRT1-mediated NF-κB inhibition also in the brain following oral dosing [144]. This effect in the brain is crucial, as it has been shown that SIRT1 controls energy metabolism, dendrite formation, axonal growth, neuronal plasticity, and neuronal endurance against stress, and suppresses inflammation by restraining NF-κB [145]. This group exerts immunomodulatory, antioxidant, and anti-inflammatory, pro-apoptotic effects and prevents DNA damage [146]. Commonly, their mechanism of action occurs through the stimulation of NRF2 protein and of its target genes *HO1*, *SOD*, and *CAT*, inflammation regression, and apoptosis inhibition via Bax and caspase-3. In high-fructose-feeding diet mice, apigenin prevents KEAP1/NRF2 binding, increases the expression and nuclear translocation of NRF2 and its downstream targets *HO-1* and *NQO1*, and in the end avoids metabolic syndrome. The PI3K/Akt pathway efficiently inhibits diabetic neuropathy evolution. In a middle cerebral artery occlusion model of male rats, it has been observed that apigenin exerts both anti-ischemic and neuroprotective properties controlling the ERK/NRF2/HO-1 pathway [147] (Figure 6). Apigenin, via PI3K/NRF2/ARE intracellular pathway system activation, impedes LPS-induced NO, iNOS, and cPLA2, thereby exerting a potential anti-inflammatory role [148].

### 10.1. Myricitrin

Myricitrin is a flavonoid that is located near the surface of the *Manilkara zapota* membrane and thus exposed to incoming radicals and, therefore, can neutralize them before they reach a reactive site in the membrane [91]. Myricitrin is the most hydrophilic flavonoid and the one with the highest ability to inhibit lipid peroxidation in phospholipid double-layer membranes [91]. Renowned for its potent antioxidant properties, myricitrin actively curbs the intracellular production of ROS, shielding against peroxide-induced toxicity (Figure 6). Myricitrin significantly increases cell survival and restores the S phase of DNA synthesis.

Its neuroprotective effectiveness is evident in its ability to safeguard against neuron damage [149,150]. In experimental studies involving Wistar rats subjected to ethidium bromide-induced hippocampal damage, myricitrin treatment decreased damage and enhanced the ability to preserve information and memories, spatial memory, balance, and anxiety [53].

### 10.2. Quercetin

Quercetin is an antioxidative and excellent neuroprotective compound, and is a promising candidate for ameliorating cognitive deficits in neurodegenerative disorders [151]. It achieves this through several mechanisms: increasing the antioxidant defense system by inhibiting the ROS/NF-κB/NLRP3 inflammasome/IL-1β and IL-18 pathways [152], as well as by upregulating the NRF2/NRF1 transcription pathway and the anti-inflammatory molecule IL-10 [153] (Figure 6).

Mirna-124 is one of the most represented miRNAs in the brain, and is important in regulating neurogenesis, neuronal differentiation, maturation, and synapse formation. Alterations of miR-124 have been found in the pathogenesis of various neurodevelopmental and neuropsychiatric disorders [154]. Furthermore, quercetin inhibits p300/CBP [155], and has shown neuroprotective properties in cellular and animal models of brain ischemia [156].

## 11. Conclusions and Future Perspectives

The rich bioactive compounds found in *Manilkara zapota* fruits are a valuable health resource, containing methyl nutrients that can modulate gene and protein expression in both the brain and peripheral tissues of offspring through epigenetic mechanisms, particularly in maternal diets. Disruptions of neuroplasticity can negatively affect neurological development. Contact with toxic elements during development, such as chronic stress, and alcohol or drug trauma, can compromise neuroplasticity and disturb normal brain development. It has proven that a combination of micronutrients enriched with different types of polyphenols and vitamins can regulate the epigenetic state of important antioxidant, anti-inflammatory, anti-apoptotic, and neuroprotective target proteins. The neuroprotective effect and epigenetic activity exhibited by the mixture of these bioactive components emphasize the synergistic relationships between these molecules during neurodevelopment, which allow the safeguarding of neonatal brain impairment and the reversal of sensory deficits, both motor and cognitive, prompted by hypoxia–ischemia.

Although *Manilkara zapota* fruit is beneficial to many, those suffering from certain conditions should consume it in moderation. In fact, subjects with latex allergies may react to *Manilkara zapota*, as it contains some common allergenic compounds. Excessive consumption of *Manilkara zapota* can cause gastrointestinal problems such as bloating or gas due to its fiber content, especially when taken in large quantities. It is advisable to gradually introduce *Manilkara zapota* into the diet to assess its tolerability, especially in children after weaning.

To date, as far as we know, there have been no comprehensive reviews addressing the role of the *Manilkara zapota* fruit in neuro-evolutionary and neurogenic epigenetic mechanisms.

Therefore, the insights discussed here could significantly improve our understanding of the nutritional and pharmacological properties of this fruit when incorporated into a diet. Future efforts should include epidemiological and clinical investigations to assess the effectiveness of *Manilkara zapota* bioactive compounds in promoting neurodevelopment and maintaining neuronal health, particularly in regions where this fruit is widely produced and consumed.

## Data Availability

References for this review were identified through search for articles from 1999 to 2024.
